# Mouthguards: does the indigenous microbiome play a role in maintaining oral health?

**DOI:** 10.3389/fcimb.2015.00035

**Published:** 2015-05-06

**Authors:** Purnima S. Kumar, Matthew R. Mason

**Affiliations:** ^1^Division of Periodontology, College of Dentistry, The Ohio State UniversityColumbus, OH, USA; ^2^Division of Biosciences, College of Dentistry, The Ohio State UniversityColumbus, OH, USA

**Keywords:** commensal, oral, health, beneficial, ecosystem, host-bacterial interactions, inter-microbial interactions

## Abstract

The existence of symbiotic relationships between bacteria and their hosts in various ecosystems have long been known to science. The human body also hosts vast numbers of bacteria in several habitats. Emerging evidence from the gastro-intestinal tract, genito-urinary tract and respiratory indicates that there are several health benefits to hosting a complex and diverse microbial community. Bacteria colonize the oral cavity within a few minutes after birth and form stable communities. Our knowledge of the oral microbiome has expanded exponentially with development of novel exploratory methods that allow us to examine diversity, structure, function, and topography without the need to cultivate the individual components of the biofilm. The purpose of this perspective, therefore, is to examine the strength of current evidence supporting a role for the oral microbiome in maintaining oral health. While several lines of evidence are emerging to suggest that indigenous oral microbiota may have a role in immune education and preventing pathogen expansion, much more work is needed to definitively establish whether oral bacteria do indeed contribute to sustaining oral health, and if so, the mechanisms underlying this role.

## Homo sapiens as a member of the bacterial kingdom

Bacteria predate humans on Earth by at least three billion years (Beraldi-Campesi, 2013); and have successfully survived the vicissitudes of drastic temperature changes, earthquakes, volcanic eruptions, and the advent of new species, evolving with each age and era. Along with their own evolution, these organisms have played a major role in shaping eukaryotic evolution, both as endosymbionts and as ectosymbionts (Pace, 1997). As *Homo sapiens* evolved, these organisms co-evolved with their host to such an extent that the human body is considered a super-organism consisting of functionally, metabolically, and spatially integrated bacterial and human cells (Ley et al., 2008). Modern-day man plays host to at least 10 times as many bacterial cells as human cells (Sleator, 2010). In fact, it might be more logical to view the human being as an inhabitant of the microbial world, rather than the reverse. Given this perspective, it is important to acquire a comprehensive understanding of the bacteria that inhabit us, and their collective genes (the human microbiome).

Recent large-scale public, private and crowd funded initiatives such as the Human Microbiome Project (HMP) (Human Microbiome Project Consortium, 2012), Metagenomics of the Human Intestinal Tract (MetaHIT) (Li et al., 2014), and UBiome (Costandi, 2013) have allowed us to explore human-microbial and inter-microbial interactions to better understand the implications of hosting our microbial fellow travelers. Through these and other studies, we are beginning to learn not only how these bacteria are acquired and their colonization dynamics, but also how diverse factors, such as host genotype, host environment and host development shape these communities (Costello et al., 2009; Zaura et al., 2009; Kumar et al., 2011; Greenblum et al., 2012; Mason et al., 2013).

## The oral microbiome as an ecosystem

The term ecosystem was introduced by Arthur Roy Clapham to describe a community of living organisms along with their living and non-living environment, interacting as a system and linked to each other through energy transfer and nutritional flow (Blew, 1996). Based on this definition, humans may be considered a collection of microbial ecosystems (Prosser et al., 2007). The body provides several habitats for colonization—the oral cavity, nasopharynx, gastrointestinal tract, vagina, and skin—each with differing topographical, nutritional, physical, and environmental characteristics. For example, the nasopharynx, gastrointestinal tract, and vagina are all non-keratinized mucosal environments with varying degrees of oxygen tension and pH levels. In contrast, the skin provides an aerobic, keratinized epithelial surface for microbial inhabitance.

The oral cavity is a unique environment in that it is divided into several smaller habitats—biotic habitats such as the non-keratinized buccal mucosa, the keratinized mucosa of the tongue and gingiva, the subgingival sulcus, and abiotic surfaces such as the enamel, dental restorations, and dental implants. At any given time, over twenty billion organisms can be found in this environment (Loesche, 1982), representing nearly 700 different species (Aas et al., 2005). Since the oral cavity is an open ecosystem, several of these species may be allochthonous members (transient visitors), however, certain organisms colonize these surfaces (autochthonous constituents) soon after birth and form organized, cooperating communities within these niches, called biofilms (Savage, 1977). It has been shown that, in certain niches (for example the tooth surface), this colonization is a very organized event with a specific temporal and spatial sequence (reviewed by Kolenbrander et al., 2006), and can be driven by environmental and host-determined factors (Mason et al., 2013). The traditional view of these biofilms is that they are comprised of species that live in equilibrium with the host immune defenses—the so-called “commensals.” However, commensalism, by definition, is a symbiotic relationship that benefits one species without harming the other. The implications of this are that the oral cavity hosts a diverse microbial community with no major benefits to the host. Since such one-sided relationships are not the norm in nature, the purpose of this perspective is to examine the currently available evidence on the health benefits of hosting a complex oral microbial ecosystem.

### Evidence for habitat specific colonization as a health benefit

A central characteristic of an ecosystem is habitat-specific colonization. For example, a wetland consists of several habitats extending from tidal creeks into low marshes and climax maritime forests, each with a specific community of flora, fauna, and microflora (Cherry, 2011). According to the physiological hypothesis, habitat specificity offers several benefits to the colonizing species, ranging from predator protection to mating to nutritional abundance (Smiley, 1978). Thus, organisms that require few host-associated benefits occupy a wide range of habitats (*generalists)*, while evolution dictates the emergence of *specialist* species that are confined to a single or narrow range of habitats. Evidence is emerging from microbial ecological systems that habitat specificity also allows a species to regulate gene expression and modify its phenotype to segregate its niche (reviewed by Young, 2006). For example, an organism determines its shape by complex algorithms that take into consideration diverse factors such as nutrient access, cell division and segregation, attachment to surfaces, passive dispersal, active motility, polar differentiation, the need to escape predators, and the advantages of cellular differentiation.

Ecologically, habitat specificity offers several benefits to the hosting species. The presence of certain algal species is important to enhance the calcifying and metabolic activities of coral-reef building anthozoans; and therefore, both species maintain their habitat specificity in all types of environments. In other marine environments, bacteria within specific habitats protect their hosts from fungal infections, detoxify host metabolites, and inhibit epibionts (White and Torres, 2009).

It is well-known that human microbial communities vary significantly by habitat. The oral microbiome is distinct from that of the gut, the ear, and the nasopharynx, even though it is geographically connected to these habitats through the esophagus, Eustachian tubes, and fauces, respectively (Frank et al., 2003; Heinemann and Reid, 2005; Flint et al., 2007; Costello et al., 2009). Within the oral microbiome, structural, spatial, functional, and compositional characteristics of supragingival and subgingival biofilms are remarkably different (Socransky and Manganiello, 1971), as are the characteristics of mucosal and tongue biofilms when compared to these tooth-related habitats (Ximenez-Fyvie et al., 2000; Socransky and Haffajee, 2005; Zaura et al., 2009). For example, *Streptococcus mitis, S. pneumoniae*, and *Granulicatella adiacens* appear to be generalists in the oral ecosystem, occupying both dental and mucosal habitats; while *Rothia dentocariosa, Actinomyces* spp., *S. sanguinis, S. gordonii*, and *A. defectiva* preferentially colonize teeth, and *Simonsiella muelleri* only colonizes the hard palate (Aas et al., 2005). Even with the same environment (supragingival or subgingival), bacterial composition varies considerably based on tooth location and site (Sreenivasan et al., 2010; Simon-Soro et al., 2013a). For example, abundances of *C. gingivalis* and *S. sanguinis* correlate with lower incisors and canines, while *Actinomyces naeslundii* 2 (also known as *A. oris*) demonstrates a positive association with upper anteriors (Haffajee et al., 2009).

While evidence demonstrates the existence of habitat-specific microbial communities in the oral cavity, the benefits conferred by this spatial segregation to community members and the implications of this phenomenon for oral health have not been as well-studied. Most of our current knowledge comes from investigations of specific species, for example, *Porphyromonas gingivalis* (iron availability and anaerobiosis), *Fusobacterium nucleatum* (pH, anaerobiosis, etc.) and oral *Streptococci* (salivary glycans, simple carbohydrates, etc.). Investigating the effect of spatial segregation on community membership and function would be critical to elucidating the role played by distinct bacterial consortia in the etiology of site-specific diseases such as caries and periodontal disease.

### Evidence for colonization resistance as a health benefit

One of the most important benefits a resident microbial community can offer to the host is resistance to invasion. In environmental ecology, invasion is defined in the process by which an exogenous species establishes itself within a resident community (Shea and Chesson, 2002). However, many human diseases are polymicrobial infections, sometimes occurring due to an overgrowth of opportunistic resident species, suggesting that “pathogens” are already present in a health-compatible environment. Hence, in human microbial ecosystems, several lines of evidence have demonstrated that the role of indigenous bacteria in controlling pathogenic colonization is by preventing pathogen expansion rather than by retarding exogenous acquisition (van der Waaij et al., 1971; Winberg et al., 1993; Drenkard and Ausubel, 2002; Wardwell et al., 2011). Disruption of resident communities with antibiotics is consistently associated with increased colonization by pathogenic species or pathologic overgrowth of certain commensals, leading to disease (Pavia et al., 1990; Pepin et al., 2005; Adams et al., 2007). This effect has been seen in the gut, vagina, and oral cavity (van der Waaij et al., 1971; Winberg et al., 1993; Ubeda et al., 2010). In certain cases, loss of colonization resistance can lead to take-over of the community not only by pathogenic bacteria, but also by higher order organisms, for example *Candida*, in both the vagina and the oral cavity (Budtz-Jörgensen, 1990; Spinillo et al., 1999). On the other hand, replenishing the resident microbiome using probiotics has been shown to reverse the effects of antibiotic-induced pathogen disease in the urinary tract, gut, and the dentition (Madden et al., 2005; Whorwell et al., 2006; Amdekar et al., 2011; Culp et al., 2011). Also, recent evidence from fecal biotherapy studies have demonstrated that restoring a native commensal population has been able to reverse pathogenic *Clostridium difficile* infection (Gough et al., 2011).

Although early evidence from non-microbial ecosystems indicated that highly diverse communities (as defined by those with more types of species) resisted exogenous invasion better than communities with fewer species (Fargione and Tilman, 2005), evidence has been emerging since then to indicate that species abundance (that is, the relative levels of each species within the community) plays a very important role, in some instances, a greater role than does species-richness (Kumar et al., 2006, 2011; Griffen et al., 2012). The first line of defense in colonization resistance is niche saturation, an ecological phenomenon where a certain number of species dominate the community, and resist colonization by pathogenic organisms (Brockhurst et al., 2007). This saturation phenomenon helps create a barrier for exogenous colonization (van der Waaij et al., 1971), prevent pathogen expansion (Gao et al., 2014), and maintains community stability, resulting in mucosal health (Abt and Pamer, 2014).

Change in species diversity is a hallmark of many bacterial dysbiotic conditions; certain diseases like bacterial vaginosis (Fredricks et al., 2005; Oakley et al., 2008), are associated with increase in diversity, while some others, for example, respiratory tract infections (influenza and bacterial pneumonia), and certain gut infections (*H. pylori* and *C. difficile)* are associated with decreased diversity. Interestingly, within the oral ecosystem, while periodontal diseases are associated with an increase in diversity (Loe et al., 1965; Listgarten, 1976; Loesche and Syed, 1978), dental caries is associated with a decrease in diversity (Simon-Soro et al., 2013b). Thus, any deviation from the stringently controlled diversity that is associated with health appears to result in disease.

In summary, literature is emerging in the gut, respiratory, urinary, and vaginal microbiomes supporting the role of colonization resistance as a health benefit. These lines of evidence include (i) pathogen colonization resulting from loss of resident microflora following antibiotic therapy, (ii) reversal of pathogen colonization by probiotic use, (iii) pathogen acquisition following changes in indigenous diversity, and (iv) reversal of pathogenic colonization following bacterial remediation (fecal transplants). Within the oral cavity, the lines of evidence have not been as robust or defined; possibly because bacteria implicated in the etiology of periodontal diseases and dental caries are already present within the health-compatible microbiome (pathobionts) (Jiao et al., 2014). Thus, the role of the oral microbiome in maintaining health may be more to prevent pathogen expansion rather than preventing exogenous acquisition. It is important to recognize the uniqueness of this ecosystem, and target research toward examining the implications of microbial homeostasis in an open, polymicrobial ecosystem.

### Importance of temporal stability, resistance, and resilience in health

In any ecosystem, three factors contribute longitudinally to health—the ability of the ecosystem to maintain its diversity, structural, and functional framework, as well as its ability to rebound from episodes of disturbance. In the pharyngeal microbiome, loss of temporal stability has been suggested the most proximal cause for the development of respiratory tract infections (Gao et al., 2014). Gao et al. have reported that patients with cystic fibrosis (CF) are the most susceptible to secondary infections, followed by chronic obstructive pulmonary disease (COPD) and asthma. Interestingly, the levels of *Bacteroidetes* were found to be lowest in CF, followed by COPD and asthma. Thus, it is hypothesized that as the protective “cover” offered by *Bacteroidetes* decreases, pathogenic *Proteobacteria* expand from their normal niche in the oropharynx and advance down the respiratory tract; their habitat-specificity being altered by the lack of competition in the “new” niche.

Oral bacteria are acquired at birth, and their colonization in the pre-dentate infant is dependent both on host genotype and on nutrition (van Steenbergen et al., 1997; Kobayashi et al., 2008). Following the development of dentition, a stable microbiome is acquired that persists into adulthood. There is evidence that bacterial composition remains stable over long periods of time (Rasiah et al., 2005; Kumar et al., 2006), even following routine dental prophylaxis and recolonization (reviewed by Teles et al., 2013). Less is known about resilience of oral bacterial communities. We have recently demonstrated that subgingival and marginal biofilms return to nearly 90% of their original compositional structure following repeated episodes of gingivitis (Joshi et al., 2014) in never smokers, but that this resilience is lost in current smokers. The host response to this “newer” microbiome is a higher than before pro-inflammatory response, suggesting that repeated episodes of gingivitis in smokers may present a higher risk for disease than in nonsmokers.

### The role of bacterial cooperativity in resistance, stability, and resilience of microbial communities

Colonization resistance and temporal stability are mediated through several inter-bacterial and host-bacterial interactions. This section will focus on what we currently know about how bacterial interactions allow species to selectively colonize, survive, and thrive in a habitat. Bacteria within human ecosystems depend upon each other for structural and metabolic cooperativity; a constraint that dictates their relative proportions within the community (Wintermute and Silver, 2010). This mutual symbiosis is one important factor in maintaining the abundances of genetically distinct species in a community and therefore, contributes significantly to microbial homeostasis. Bacterial colonization of a habitat begins through non-random species selection. This non-random event is facilitated by several inter-bacterial interactions, for example, nutritional syntrophy, coaggregation, antagonism, and communication.

Syntrophy or nutritional symbiosis [also known as cross-feeding (from Greek for eating together)] is one of the oldest mechanisms facilitating the formation of polymicrobial communities. Work from the gut has provided insight into the role of symbionts in shaping the evolution of microbial components of this microenvironment through lateral gene transfer. For example, gut dwelling *Bacteroidetes* have used this mechanism to vary their cell surface, sense their environment, and harvest nutrient resources present in the distal intestine (Xu et al., 2007).

In oral biofilms, this phenomenon is not as well-characterized, however, it has previously been shown that *Veillonella* and *Streptococcus, two* of the earliest and most abundant genera to colonize oral biofilms, share a nutritional syntrophy, in that the *Veillonellae* utilize the lactate that is produced by the *Streptococci* as a food source (Kuramitsu et al., 2007). Also, *Streptococcus sanguis* and *S. oralis* exhibit synergy in degrading mucins, thereby allowing efficient utilization of host glycopolysaccharides for nutrition (Van der Hoeven and Camp, 1991).

Coaggregation among the early colonizers is another important mechanism that controls the composition of tooth-associated biofilms. *Streptococci*, due to the presence of Antigen I/II receptors for salivary agglutinin glycoprotein, are the primary orchestrators of coaggregation events. Not only do they bind to salivary pellicle, dentin and collagen, but also, the presence of these receptors is essential for acquisition of another early colonizer *A. naeslundii* (Kolenbrander et al., 2006). Also, incorporation of the bridge species, *F. nucleatum* into the biofilm has been shown to be dependent on *A. naeslundii* (Periasamy et al., 2009). Recent evidence suggests an important role for *Candida* species in maintaining oral health, by providing metabolic, chemical, and physical support for colonization by certain bacteria (reviewed by Krom et al., 2014).

Antagonism is the collective ability of the normal microbiota to prevent colonization of exogenous and opportunistic pathogens. In the gut, for example, the presence of butyrate, a short chain fatty acid produced as a metabolic byproduct by some commensals, down regulates expression of virulence genes in *Salmonella* spp. (Gantois et al., 2006) The earliest reports of bacterial antagonism in the oral environment came from Hillman and Socransky, who demonstrated that plaque from periodontally healthy individuals was capable of inhibiting growth of certain periodontal pathogens (Hillman et al., 1985). Evidence has shown some commensal oral bacteria have antagonistic activity against periodontopathogens (van Essche et al., 2013). Specific examples of bacterial antagonism by means of producing metabolites in the oral cavity include hydrogen peroxide production by streptococcal species to inhibit growth of periodontopathongens (Hillman et al., 1985) and lactic acid production to prevent *Pseudomonas aeruginosa* incorporation into the biofilm (He et al., 2011). Evidence has shown *Streptococci* exhibit antagonistic properties toward certain *Staphylococci* in the oral cavity as well (Krzeminski and Raczynska, 1993). Some indigenous microbiota take colonization resistance a step farther by producing specific antibiotics, such as bacteriocin production in strains of *Streptococcus salivarius*, that act on specific pathogens to prevent their colonization of the community (Sanders and Sanders, 1982). This mechanism has also been studied in response to caries-causing bacteria (reviewed by Kreth et al., 2009). *S. sanguinis* and *S. gordonii* produce hydrogen peroxide, a chemical that decreases proliferation of *S. mutans* in a cell-density independent manner. *S. oligofermentans* utilizes the lactic acid produced by *S. mutans* to generate H_2_O_2_.

Microbes are also in direct competition for available nutrients, and in many cases the indigenous microbiota create food webs where one species end product is used by another species (Ley et al., 2006). This sequestration of nutrients by the indigenous microbiota is designed to make the colonization of non-indigenous species very difficult (Freter et al., 1983). In the gut for example, the nutrient depletion provided by the indigenous microbiota plays a role in suppressing *C. difficile* overgrowth (Wilson and Perini, 1988).

In summary, structural, metabolic, and chemical interactions between bacteria play an important role in maintaining community hemostasis by supporting the critical proportions of these species in a health-compatible microbiome. The evidence for health benefits of these interactions has been exemplified in the caries literature.

### Educating the host immune system as a health benefit

Evidence is emerging to suggest that lack of a bacterial stimulus can lead to the development of atopy, a genetic predisposition to general allergic reactions. Several mechanisms have been postulated to explain this connection, with the “Hormetic Theory” being the most widely accepted (Bukowski and Lewis, 2007). The Hormetic theory suggests that exposure to a commensal bacterial flora during early years of life serves to educate the immune system, enabling it to distinguish between pathogens and host proteins. Children with low levels of the commensals *Lactobacillus* and *Bifidobacterium* demonstrate a greater predisposition to allergies (Bjorksten et al., 2001; Sepp et al., 2005). Further, administration of prenatal *Lactobacillus GG* to mothers of high-risk infants decreased the incidence of atopy (Kalliomaki et al., 2001).

Recent evidence suggests the human microbiome is also capable of directly stimulating various components of the innate and adaptive immune responses. Much of this evidence comes from studying the gut which houses a complex and diverse microbial community (Hooper and Gordon, 2001; Ley et al., 2006; Neish, 2009). The first line of immunity to foreign species is the physical barrier of the epithelial tissue. In the gut, the gut-associated lymphoid tissue (GALT), is severely underdeveloped in germ-free mice, but undergoes enlargement when exposed to antigen (Pollard and Sharon, 1970), thus suggesting a role of host-microbe interactions in the initial development of early immunity. Additional animal studies involving germ-free model systems demonstrate greater antigen transportation compared to animals with a resident biofilm (Sudo et al., 1997). Further, germ-free animals mount a severe immune response when exposed to commensal bacteria.

### Contributions of bacteria in establishing epithelial barrier function

Colonization of a germ-free model by *B. thetaiotaomicron*, a member of the indigenous gut flora, helps establish the epithelial barrier by inducing the expression of Paneth cell proteins (Hooper et al., 2003) and a decay-accelerating factor that facilitates repair (Hooper et al., 2001). Further, the release of indole, a microbial quorum-sensing molecule, has been shown to increase expression of tight junction and adherent junction molecules in the colonic epithelial tissue (Shimada et al., 2013). The increased expression of ZO-2, a tight junction protein, has also been observed following the administration of probiotics, namely *E. coli* Nissle 1917, both *in vitro* (Zyrek et al., 2007) and *in vivo* (Ukena et al., 2007).

In the oral environment, Ye et al. characterized an increase in the expression of tight junction components in oral epithelium following binding of the normal microbiota species *S. gordonii* (Ye et al., 2013) and identified that binding of these commensals through the CD24 receptor of oral epithelial is responsible for this health associated tissue phenotype (Ye et al., 2014).

Thus, currently, there is minimal evidence in the oral cavity to indicate a role for the indigenous microbiome in affecting tissue phenotype, although emerging evidence suggests that this may an important avenue of investigation.

### Contributions of bacteria to developing TLRs

An important link between microbes and epithelial cells in innate immunity is Toll-like receptors (TLRs). Evidence suggest that the expression of TLRs on gut epithelial cells is decreased in germ-free mice when compared to mice with conventional microbiota (Lundin et al., 2008). TLRs respond to both commensals and pathogens, but evidence now suggests TLRs interaction with commensals contributes to intestinal epithelial homeostasis and protection from injury (Rakoff-Nahoum et al., 2004). The location of TLR expression in the gut epithelium has been suggested to play a role in the hosts ability to tolerate commensals and mount a more targeted inflammatory attack against pathogens (Furrie et al., 2005). Evidence also suggests that a benefit of probiotics for maintaining a healthy gut works through the TLR9 pathway, which mediates the anti-inflammatory effects observed with probiotic use (Rachmilewitz et al., 2004). The activation of TLR2 in a stress-induced inflammation model also suppressed mucosal inflammation in the gut by promoting tight junction integrity in the epithelial barrier (Cario et al., 2007). On the skin, commensal *Staphylococcal* species inhibit skin inflammation through the regulation of TLR3 by *Staphylococcal* lipoteichoic acid (LTA) activation of a TLR2-dependent pathway (Lai et al., 2009). Subsequently, the activation of TLR2 by *Staphylococcus epidermis* induces keratinocyte expression of antimicrobial peptides to enhance innate immunity toward pathogens (Lai et al., 2010).

### Bacteria and neutrophil function

The cellular components of the innate immune system primarily include neutrophils. Evidence suggests the recruitment of neutrophils to tissue is increased by the presence of the mircobiota (Kanther et al., 2014). Similarly, work from Zenobia et al. has provided evidence that commensal bacteria in the oral cavity selectively upregulate CXCL2 expression leading to an increase in neutrophil recruitment to “prime” healthy gingival tissue (Zenobia et al., 2013). Interleukin-8 is another important chemokine in the innate immune pathway known to attract neutrophils and enhance phagocytosis. The normal microbiota has been shown to induce IL-8 (Darveau et al., 1998; Vankeerberghen et al., 2005), presumably to recruit neutrophils to a potential pathogen colonization site to help prevent overgrowth of pathogens. Not only does the commensal microbiota play a role in recruiting neutrophils, but evidence from a germ-free (Clarke et al., 2010) model suggests that a lack of commensal microbiota reduces phagocytosis and antimicrobial killing activity (Ohkubo et al., 1990, 1999). Commensal bacteria also induce low-level expression of human beta defensins, presumably to keep the innate immune system in a limited activation state (Vankeerberghen et al., 2005) and have been implicated in the regulation of gene expression of the complement system, another important arm of innate immunity (Chehoud et al., 2013).

In summary, a large body of evidence suggests that the host associated microbiome plays an important role in regulating the innate immune system, through epithelial bacterial function, bacterial recognition pathways and signals and innate immune cell functions. However, a similar level of evidence is currently lacking in the oral microbiome.

### Bacteria and regulatory T cell education

Although significant evidence exists supporting the role the indigenous microbiota plays in fortifying innate immunity, evidence continues to grow linking commensal microbiota to the adaptive immunity cascade of events. The peripheral education of regulatory T cells (Treg) in the colon by antigens derived from the commensal microbiota ensures the local tolerance of this microbial community by the host (Lathrop et al., 2011). One well-identified human commensal, *Bacteroides fragilis*, directs Treg cell education using the immunomodulatory molecule, polysaccharide A (PSA) (Round and Mazmanian, 2010). PSA induces an IL-10 response in T cells that inhibits Th17 expansion preventing future mucosal damage (Round et al., 2011). Similar Treg cell induction and anti-inflammatory effects are seen with colonization of *Clostridia* species (Atarashi et al., 2011, 2013; Chiba and Seno, 2011; Nagano et al., 2012). In contrast, colonization of the gut intestinal tissue by segmented filamentous bacteria leads to an increase in mature Th17 cells and Th1 cells (Gaboriau-Routhiau et al., 2009; Ivanov et al., 2009).

Similar evidence exists in the oral cavity where members of commensal oral bacteria prime dendritic cells for Th2 and Treg differentiation (Kopitar et al., 2006). Shin et al demonstrated that *F. nucleatum*, an oral commensal, induces Th1 and Th3 immune responses, while *Treponema denticola*, a pathogenic species, induced a Th1-dominant response (Shin et al., 2013).

### Bacteria and B cell education

Lastly, evidence indicates that commensal microbiota play a role in B cell development and maturation. In human infants, maturation of the mucosal defense system, particularly cells that secrete IgA and IgM, is dependent on the presence of a normal gut flora (Klaasen et al., 1993; Gronlund et al., 2000). This suggests that the development of immune tolerance is dependent on early and sustained exposure to a stable biofilm. Recent evidence suggests that B cell maturation may in fact be dependent on intestinal bacterial colonization as mice colonized early with *E. coli* and *Bifidobacteria* have an increased population of CD20+B cells expressing memory marker CD27 (Lundell et al., 2012). Following development of the lymphoid tissue, the host immune system faces the challenge of finding balance between mounting a swift response toward invading pathogens, but controlling that response against commensals. One way the host controls this response in mice is by using dendritic cells to “sample” the gut lumen and keep live commensals engulfed for a few days to selectively induce IgA production (Macpherson and Uhr, 2004). This localized production of IgA helps maintain immune system homeostasis and allows the human host to distinguish between a commensal and pathogenic colonization providing the necessary defense mechanisms to control an infection.

### Other benefits

Evidence is emerging to suggest that oral bacteria may play a critical role in nitric oxide NO homeostasis (Kapil et al., 2013; Hyde et al., 2014). They do so by reducing dietary nitrate to bioactive NO, a critical symbiotic relationship since humans lack the enzymes to carry out this function. The effects of NO in maintaining cardiovascular integrity are well-established in literature. Thus, recent studies point to a cardio-protective role for oral bacteria; and may provide a critical link in the oral-systemic health connection.

Thus, although the traditional view of an indigenous microbiome is one that provides a nondestructive inflammatory stimulus to the host, thereby ensuring host-bacterial equilibrium, emerging evidence indicates that this community appears to play an active role in developing the host innate immunity and priming the adaptive immune response.

## Future steps

The role of the microbiome in maintaining health in several human ecosystems is an emerging and exciting field of study. While the health benefits of supporting a large microbial community are actively being explored in reference to the gut, genito-urinary tract, and respiratory system; similarly robust evidence is lacking in relation to the oral microbiome. Several decades of research have been focused on exploring the microbiota associated with oral diseases. While the importance of this quest cannot be downplayed, it is sometimes easy to forget that the ultimate aim of treating disease is to restore health, and the only successful method of preventing disease is by maintaining health. Hence, it is important to focus on the health benefits provided by our microbial fellow travelers and to expend some effort in cataloging and characterizing not only this community, but also the host determinants that play a role in shaping this population.

### Conflict of interest statement

The authors declare that the research was conducted in the absence of any commercial or financial relationships that could be construed as a potential conflict of interest.
